# Repression of *Sex4* and *Like Sex Four2* Orthologs in Potato Increases Tuber Starch Bound Phosphate With Concomitant Alterations in Starch Physical Properties

**DOI:** 10.3389/fpls.2018.01044

**Published:** 2018-07-23

**Authors:** Ebrahim Samodien, Jonathan F. Jewell, Bianke Loedolff, Kenneth Oberlander, Gavin M. George, Samuel C. Zeeman, Fred F. Damberger, Christell van der Vyver, Jens Kossmann, James R. Lloyd

**Affiliations:** ^1^Department of Genetics, Institute for Plant Biotechnology, Stellenbosch University, Stellenbosch, South Africa; ^2^Department of Conservation Ecology and Entomology, Stellenbosch University, Stellenbosch, South Africa; ^3^Institute of Molecular Plant Biology, ETH Zürich, Zürich, Switzerland; ^4^Institute of Molecular Biology and Biophysics, ETH Zürich, Zürich, Switzerland; ^5^Biomolecular NMR Spectroscopy Platform, Department of Biology, ETH Zürich, Zürich, Switzerland

**Keywords:** starch phosphate, starch phosphatase, RVA, chain length distribution, cold sweetening, starch degradation

## Abstract

To examine the roles of starch phosphatases in potatoes, transgenic lines were produced where orthologs of *SEX4* and *LIKE SEX FOUR2* (*LSF2*) were repressed using RNAi constructs. Although repression of either *SEX4* or *LSF2* inhibited leaf starch degradation, it had no effect on cold-induced sweetening in tubers. Starch amounts were unchanged in the tubers, but the amount of phosphate bound to the starch was significantly increased in all the lines, with phosphate bound at the C6 position of the glucosyl units increased in lines repressed in *StSEX4* and in the C3 position in lines repressed in *StLSF2* expression. This was accompanied by a reduction in starch granule size and an alteration in the constituent glucan chain lengths within the starch molecule, although no obvious alteration in granule morphology was observed. Starch from the transgenic lines contained fewer chains with a degree of polymerization (DP) of less than 17 and more with a DP between 17 and 38. There were also changes in the physical properties of the starches. Rapid viscoanalysis demonstrated that both the holding strength and the final viscosity of the high phosphate starches were increased indicating that the starches have increased swelling power due to an enhanced capacity for hydration.

## Introduction

Starch is one of the plant products most extensively used by industry and the amount of starch isolated within Europe alone was approximately 10.7 million tons in 2016^1^. It is a polyglucan consisting of two fractions; branched amylopectin and lesser branched amylose. Most starch used in industry is isolated from seeds of cereals, such as maize or wheat, with smaller, yet significant amounts coming from plants with underground storage organs, such as potato or cassava (Zeeman et al., 2010). Although potato starch only makes up approximately 10% of the total market, its physicochemical properties make it useful in specific industries, such as the production of noodles, because it forms a particularly viscous gel when heated in water (Lu et al., 2012). The high viscosity is caused by the relatively large amount of covalently bound phosphate present in potato starch (Lorberth et al., 1998), which is virtually absent in starches isolated from cereal endosperm (Jane et al., 1996; Kasemsuwan and Jane, 1996; Blennow et al., 2000). Understanding starch (phosphate) metabolism and using this to increase starch granule bound phosphate is, therefore, of industrial importance.

Starch phosphate is present at the C6- and C3-positions of the constituent glucosyl residues of amylopectin and is incorporated by two related enzymes: glucan, water dikinase (GWD) and phosphoglucan, water dikinase (PWD). GWD specifically phosphorylates the C6-position while PWD is responsible for incorporation at the C3-position (Ritte et al., 2002, 2006), but only in starch which has been previously phosphorylated by GWD (Kötting et al., 2005). Repression of either GWD or PWD has been demonstrated to decrease starch bound phosphate in a number of plant species (Mahlow et al., 2016), while elevating GWD leads to increased amounts (Carciofi et al., 2011; Chen et al., 2017; Xu et al., 2017a).

The phosphate is removed by two proteins, SEX4 and LIKE SEX FOUR2 (LSF2). SEX4 was originally identified as being involved in starch metabolism through study of a mutation that impairs starch mobilization in Arabidopsis leaves (Niittyla et al., 2006; Sokolov et al., 2006), while LSF2 was examined due to its protein sequence similarity to SEX4 (Santelia et al., 2011). Both of these proteins catalyze removal of the phosphate from amylopectin and starch granules, as well as from phosphorylated malto-oligosaccharides that are the product of amylolytic starch breakdown (Gentry et al., 2007; Kötting et al., 2009; Santelia et al., 2011). SEX4 has a preference for removing phosphate at the 6-position, while LSF2 removes phosphate mainly at the 3-position of glucose moieties (Kötting et al., 2009; Hejazi et al., 2010; Santelia et al., 2011; Meekins et al., 2015). One other locus in Arabidopsis (*LSF1*), that shows similarity to both SEX4 and LSF2, is not thought to encode a starch phosphatase (Comparot-Moss et al., 2010) as several attempts to measure its activity have been unsuccessful (Silver et al., 2014). Interestingly, expression of a mammalian Laforin like polyglucan phosphatase, which is similar in both structure and function to SEX4 and LSF2 (Gentry et al., 2007), increased potato starch granule bound phosphate, although the reasons for this are unclear (Xu et al., 2017b).

Work in Arabidopsis has suggested a new strategy to increase starch phosphate in organs that store starch. Mutations in *AtLSF2*, but not *AtSEX4*, led to increases in starch bound phosphate in Arabidopsis leaves (Santelia et al., 2011). *Atsex4* plants, on the other hand, accumulate phospho-oligosaccharides (Kötting et al., 2009; Santelia et al., 2011). It is, therefore, possible that repression of orthologs of these starch phosphatases could lead to increases in starch phosphate in storage organs of other species, such as potato tubers, however, this has never been tested.

The elucidation of the pathway of starch degradation in leaves has made great progress over the past two decades, especially in Arabidopsis where roles for SEX4 and LSF2 have been determined (Stitt and Zeeman, 2012). It remains to be seen, however, if the pathway established in leaves is conserved in other plant organs. When potato tubers are stored at low temperatures the starch within them is broken down leading to the accumulation of reducing sugars (Müller-Thurgau, 1882). This is detrimental to the potato industry as the sugars react with amino acids when the tuber is fried, leading to discoloration alongside production of the neurotoxin acrylamide (Mottram et al., 2002; Stadler et al., 2002). Repression of the orthologous *SEX4* or *LSF2* genes in potato may help solve the problem of cold-induced sweetening in tubers. In this study, we produce transgenic potatoes using RNAi constructs designed to repress orthologs of either *SEX4* or *LSF2*. In both cases, starch phosphate in the plants increased, but there was no repression of cold-sweetening. The increased phosphate was accompanied by other changes in starch structure and with alterations in its gelling properties.

## Materials and Methods

### Production of RNAi Constructs and Plant Transformation

Arabidopsis SEX4 (UniprotKB/Swiss-Prot: Q9FEB5.1) and LSF2 (UniprotKB/Swiss-Prot: Q9SRK5.1) peptide sequences were used to identify tobacco expressed sequence tags (EST’s) (*NtSEX4*: DV158006.1; *NtLSF2*: DV162318.1) using the tBLASTn algorithm at the NCBI^2^. Plasmids containing EST’s corresponding to each ortholog were obtained from the French Plant Genomic Resource Center and 300 bp fragments were amplified by PCR using the following primers: [NtSEX4FWD, 5′CAGGGCCCCGTGCCGAAATAAGGGATT3′ (*Sma*I site underlined); NtSEX4REV: 5′GAGTCGACTGTCAATGTGGCAGGCTTGG3′ (*Sal*I site underlined; NtLSF2FWD: 5′GAGATATCATGTTCAGAGCTTGGGAATTC3′ (*Eco*RV site underlined); NtLSF2REV: CAGGATCCTTTTTAGCCTTTTCATAAGTAGCT3′ (*Bam*HI site underlined)]. The *NtSEX4* and *NtLSF2* sequences are sufficiently similar in sequence to be effective in strategies designed to silence their putative potato orthologs [Accession numbers DQ241838 (*StSEX4*) and XM_006361255 (*StLSF2*)]. Amplicons were restricted with *Sma*I and *Sal*I or *Eco*RV and *Bam*HI before being ligated into the same sites of pBluescript-SK+ (Agilent Technologies Inc.). RNAi constructs were produced in the plant transformation vector pHellsgate2 (Wesley et al., 2001) by incubating with BP Clonase (Invitrogen) and DNA that had been PCR amplified from the vectors using the following primers: [T7AttB1: 5′GGGGACAAGTTTGTACAAAAAAGCAGGCTGTAATACGACTCACTATACGGC3′ (AttB1 site underlined) and T3AttB2 5′GGGGACCACTTTGTACAAGAAAGCTGGGTAATTAACCCTCACTAAAGGG3′ (AttB2 site underlined)]. The silencing vectors were transformed into *Agrobacterium tumefaciens* GV2260 by the freeze-thaw method (Höfgen and Willmitzer, 1988) and used to manufacture transgenic potato (*Solanum tuberosum* L. var Désirée) plants by the method of Dietze et al. (1995).

### Phylogenetic Analysis

The respective tobacco ESTs and potato sequences were translated into protein sequences and aligned with protein DSP domain sequences reported previously (Santelia et al., 2011). Expressed sequence tags (ESTs) for tobacco were obtained from the French Plant Genomic Resource Center^3^ (Accession numbers: *SEX4* and *LSF2* as above; *LSF1*, EB448369). Potato sequences were obtained from the Potato Genome Sequencing Consortium^4^ (*SEX4* - PGSC0003DMT400070294 CDS*; LSF1*- PGSC0003DMT400077364 CDS; *LSF2* - PGSC0003DMT400074765 CDS). The aligned matrix contained 71 taxa and 155 amino acid characters, of which 148 were parsimony-informative. Parsimony analysis was performed using the PAUP^∗^ V 4.0 10 computer program (Swofford, 2002). Heuristic searches (100 replicates) using stepwise-addition for starting trees and TBR branch-swapping found a single island of 110 most parsimonious trees of length 1985. Node support was measured using the parsimony bootstrap (100 replicates) with the same settings as the initial heuristic search.

### Growth of Plants and Sampling

Potatoes were planted from tissue culture into 30-cm diameter pots containing a mix of 50% (v/v) sand 50% (v/v) potting mix and were grown in a glasshouse. Leaf samples for starch and sugar analysis were taken from 10-week-old plants over a diel cycle using a cork borer and were immediately frozen in liquid N_2_ and stored at -80°C until analysis. Tubers were harvested from 4-month-old plants and weighed individually. At this point core samples were taken using a cork borer and frozen in liquid N_2_. These were stored at -80°C before either RNA or proteins were isolated, or carbohydrate contents determined. The rest of the tubers were either used for extraction of starch by the method of Edwards et al. (1995) using the modifications of Lloyd et al. (1999), or were stored at 4°C for 8 weeks after which samples were taken (as described above) for analysis of starch and reducing sugar amounts.

### Determination of Repression of Gene Expression

Total RNA was isolated from tuber and leaf material using the Plant RNeasy Mini kit (Qiagen, South Africa), as described by the manufacturer. The complementary DNA (cDNA) template was obtained *via* reverse transcription of 1 μg of total RNA, using an oligo (dT_15_) primer and M-MLV (H-) reverse transcriptase (Promega, Anatech, South Africa) according to the manufacturer’s instructions. The semi quantitative-PCRs (sq-PCR) were conducted with GoTaq^®^ DNA polymerase (Promega) in a 50-μl reaction (3 μl cDNA, 1.25 U DNA polymerase, 5× green PCR buffer, 0.5 mM of each dNTP, and 0.5 μmol of each primer) for 24 cycles, with a primer annealing temperature optimum of 58°C for all respective sq-PCR primer pairs. The constitutively expressed gene, β*-tubulin2* (Nicot et al., 2005; NCBI accession number 609267), was used to determine the number of cycles used for the sqRT-PCR, where expression occurred in the linear range. The *St*β*-tubulin2, StLSF1*, *StLSF2*, and *StSEX4* primer pairs (StTUBFWD 5′ATGAGAGAAATCCTTCACATTC, StTUBREV 5′ GTCAGACACCTTTGGAGAAG; StLSF1FWD 5′ ATGTGCGTAATGGCCTC, StLSF1REV 5′ CCCCAGTTGAAGAGTCACTG; StLSF2FWD 5′ ATGAGAGCTCTCTGGAACTCC, StLSF2REV 5′ CCTTTTCCTTCTGAAATCGC; StSEX4FWD 5′ ATGAATTGCCTTCAGAATCTTC, StSEX4REV 5′ TGATGGCATTGTTCAGTAGT) were designed to amplify fragments of 0.5 kb from the cDNA.

### Starch and Sugar Amounts

Starch and soluble sugar amounts were determined enzymatically from tuber or leaf tissues by the method of Müller-Röber et al. (1992). Leaves were kept in the dark by covering with aluminum foil, and starch assessed semi quantitatively by staining with Lugol’s solution using the method of Scheidig et al. (2002).

### Starch Parameters

Amylose was estimated using an iodine binding assay (Hovenkamp-Hermelink et al., 1988) while the concentration of glucose 6-phosphate in starch was determined enzymatically by the method of Nielsen et al. (1994).

Glucose 3-phosphate content was calculated from that figure using the ratio of C6:C3 bound phosphate in each of the transgenic lines determined by ^31^P NMR. NMR samples contained 50 mg starch digested with α-amylase and amyloglucosidase, D_2_O was added (10% v/v) and the pH adjusted to 6.0 with 0.2 M NaOH as described by Santelia et al. (2011). Measurements were conducted at 30°C using an Avance III 600 MHz spectrometer equipped with a QCI CryoProbe (Bruker). One-dimensional ^1^H and ^31^P spectra were measured for each sample. ^31^P 1D spectra were accumulated in multiple experiments for a total acquisition times of 1.2 h per sample.

Chain length distributions were analyzed by boiling 20 μg of purified starch in water followed by debranching using isoamylase, as described by Streb et al. (2008). Linear glucans were then dephosphorylated using Antarctic Phosphatase (New England Biolabs) as described previously (Kötting et al., 2009). The resulting glucans were briefly boiled and centrifuged at 16,000 *g*. Linear glucans in the supernatant were separated and measured by HPAEC-PAD using a Dionex PA-200 column.

Swelling power was determined by the method of Howard et al. (2014). Gelling properties were recorded in a RVA4500 (Perten Instruments) by placing a 16% (w/v) starch/water slurry in the instrument. The slurry was stirred for 10 s at 960 rpm and then at 160 rpm for the remaining time. The temperature profile was as follows: 50°C for 1 min, followed by a linear increase to 95°C over 3 min 42 s, hold at 95°C for 2 min 30 s, cool to 50°C over 3 min 48 s and hold at 50°C for 2 min. The viscosity was recorded continuously during this time. Normally there is an initial quick increase in viscosity to a maximum (Peak viscosity), before reducing to a trough (Trough viscosity) after which it can increase again to the final viscosity. The difference between peak and trough viscosities is known as breakdown while the difference between the trough and final viscosities is the setback.

Starch granule size was determined using a Saturn DigiSizer 5200 particle size analyzer. Five grams of starch was suspended in MilliQ water within the machine and directed toward a 30 mW, 687 nm solid-state laser. Starch granule size distributions were determined based upon the amount of light scattered by each particle using a refractive index value of 1.54, as outlined by Cledat et al. (2004).

### Scanning Electron Microscopy

Isolated starch granules were sprinkled onto double sided tape and coated in gold using a sputter coater (s150A, Edwards). Micrographs were taken in a scanning electron microscope (Zeiss Evo MA15VP) at an accelerating voltage of 25 kV.

### Immunoblots and Activity Gels

Proteins were extracted from tuber material by grinding in 50 mM TRIS-HCl (pH7.0), 5 mM EDTA, 5 mM MgCl_2_, 1 mM β-mercapoethanol, 0.1% (v/v) TWEEN-20 and 1 mM PMSF. Following centrifugation, the supernatant was isolated, and the amount of protein determined using a Bradford assay kit (Bio-Rad).

For immunoblots 25 μg of crude protein extract was denatured by heating at 95°C for 5 min in 2% (w/v) SDS, 10% (v/v) glycerol, 60 mM TRIS-HCl (pH 6.8) before being separated by either 8 or 10% (w/v) SDS-PAGE. Separated proteins were blotted onto nitrocellulose membranes using a semi-dry blotter (Bio-Rad). Immunoblots were performed using antibodies that specifically detect GWD (Lorberth et al., 1998), PWD (Kötting et al., 2005), or SEX4 (Niittyla et al., 2006).

Enzyme activities were examined by separating 100 μg of crude protein extract on 8% (w/v) polyacrylamide gels containing either 0.3% (w/v) oyster glycogen (for starch synthase) or 0.02% (w/v) oyster glycogen (for starch branching enzyme and starch phosphorylase) for 3 h at 4°C at 10 V cm^-1^. For starch synthase the gel was incubated in 50 mM Tricine-NaOH (pH 8.5), 0.5 M trisodium citrate, 2 mM EDTA and 2 mM DTT for 30 min at 4°C before the buffer was removed and replaced with the same buffer, but containing 1 mM ADP-glucose in addition. This was left at room temperature overnight and starch synthase activity revealed through staining with Lugol’s solution. For starch branching enzyme and starch phosphorylase two gels were incubated in 50 mM HEPES-NaOH (pH7.0) 10% (v/v) glycerol of 30 min at 4°C. This buffer was removed and replaced with the same buffer containing 2.5 mM AMP and 50 mM glucose 1-phosphate for one gel and 2.5 mM AMP, 50 mM G1P and 28 units of phosphorylase a (from rabbit muscle) for the other. Both the gels were incubated at room temperature overnight and activities were revealed by staining with Lugol’s solution. Bands appearing in both gels are formed by starch phosphorylase, while bands appearing only in their gel incubated with phosphorylase a are formed by starch branching enzyme.

### Data Analysis

One-way analysis of variance and *post hoc* Bonferroni–Holm partitioning were determined in Microsoft Excel 2010 using Daniel’s XL Toolbox add-in (Kraus, 2014).

## Results

### Repression of *SEX4* and *LSF2* Orthologs

It has been shown previously that it is possible to separate SEX4, LSF1, and LSF2 orthologs into three clades (Santelia et al., 2011), although the potato orthologs were not included in that analysis. Therefore, potato genes that showed high similarity to *AtSEX4*, *AtLSF1*, or *AtLSF2* were identified through tBLASTn searches of the potato genome (Xu et al., 2011) using the amino acid sequence of the Arabidopsis proteins. These were combined with sequences from several species used for the previous analysis (Santelia et al., 2011) to construct a new phylogenetic tree (**Supplementary Figure S1**). The potato proteins fell into each of the clades indicating that the three proteins identified in Arabidopsis are conserved in potato and are likely to have the same biochemical function. The predicted protein sequences from the potato genes were similar in structure to the equivalent Arabidopsis proteins (Santelia et al., 2011) in that StSEX4 contains both a dual specificity phosphatase (DSP) domain and a starch binding domain, while StLSF2 contains only a DSP domain.

**FIGURE 1:**
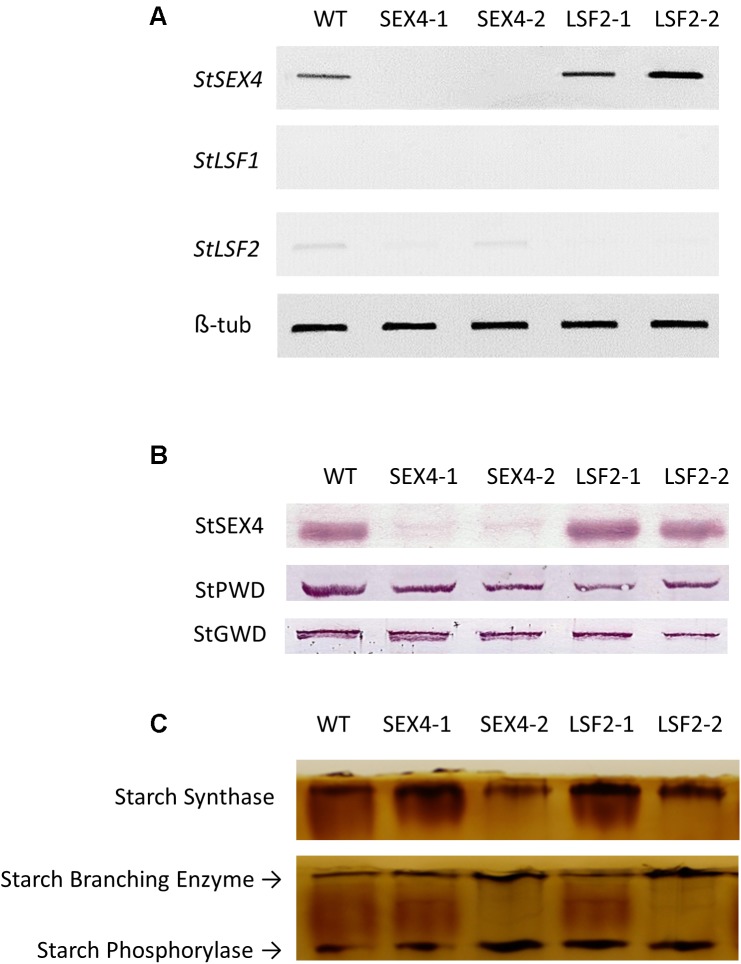
Accumulation of *StSEX4, StLSF1*, and *StLSF2* transcript, analysis of StSEX4, StPWD, and StGWD protein amounts and examination of the main starch polymerizing activities in tubers of the transgenic lines. **(A)** Semi quantitative RT-PCR of orthologs of *StSEX4* and sex-four like genes and **(B)** protein amounts of StSEX4, StPWD, and StGWD as determined by immunoblotting using specific antibodies. Equal amounts (25 μg) of crude protein extracts from freshly harvested tubers were separated by SDS-PAGE and blotted onto nitrocellulose membranes prior to immunoblotting. **(C)** Zymogram analysis of starch synthase, starch branching enzyme and starch phosphorylase. Equal amounts (100 μg) of crude protein extracts were separated by native polyacrylamide gel electrophoresis prior to detection of enzyme activities.

**FIGURE 2:**
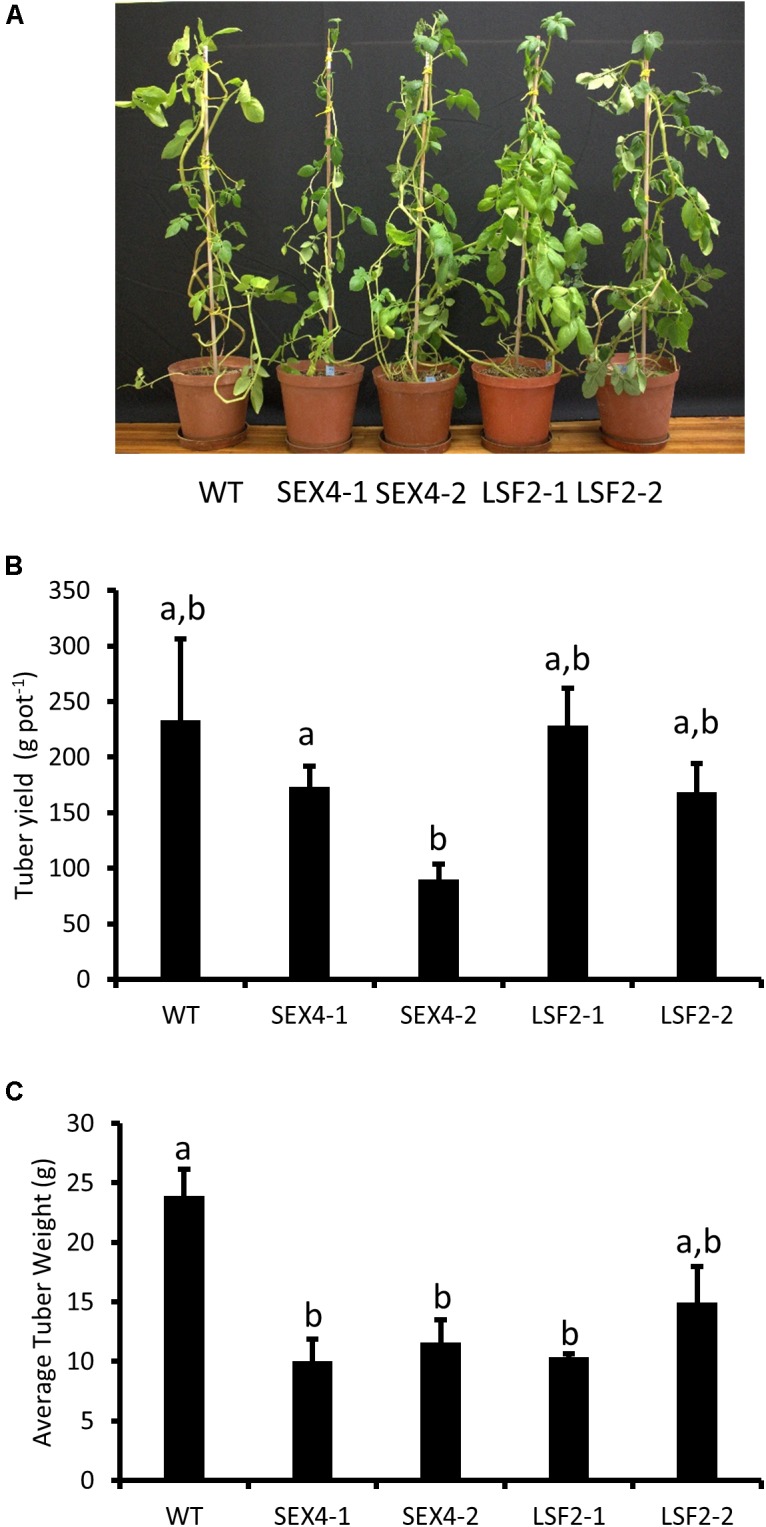
Analysis of transgenic plants. Pot trials were used to assess **(A)** plant morphology after 10 weeks growth, **(B)** tuber yield per plant, and **(C)** average tuber size. Data represents mean ± SEM of measurements from at least four pots. Letters represent groups with similar means at the 5% significance level as determined using the Bonferroni–Holm *post hoc* test following a one-way analysis of variance.

RNAi constructs designed to repress either *StSEX4* or *StLSF2* were transformed into potato and plants were selected based on decreased expression of the respective genes in growing tubers, determined by semi-quantitative RT-PCR (**Figure 1A**). Repression of *StSEX4* appeared to also repress *StLSF2* in one line (SEX4-1), however we were unable to detect expression of *StLSF1* in growing tubers. Immunoblots showed that repression of *StSEX4* led to a decrease in StSEX4 protein accumulation, but we were unable to examine StLSF2 protein abundance due to the lack of an appropriate antibody. Repression of neither *StSEX4* nor *StLSF2* genes altered the amounts of the starch phosphate incorporating enzyme, GWD (**Figure 1B**). However, PWD amounts appeared to be slightly decreased in all the transgenic lines (**Figure 1B**). In gel assays were used to examine the activities of enzymes known to affect starch polymerization. There were no consistent differences in starch synthase, starch branching enzyme or starch phosphorylase activities in crude extracts from tubers of the transgenics lines, compared with the controls (**Figure 1C**). The transgenic plants were phenotypically unaltered from each other or from the untransformed control (**Figure 2A**). Although there were some differences in tuber yield between the transgenic lines, this was not consistent within different lines where the same gene was repressed (**Figure 2B**), however, the tubers in three of the four transgenic lines were smaller than the untransformed control (**Figure 2C**).

### Effects on Starch Degradation

**FIGURE 3:**
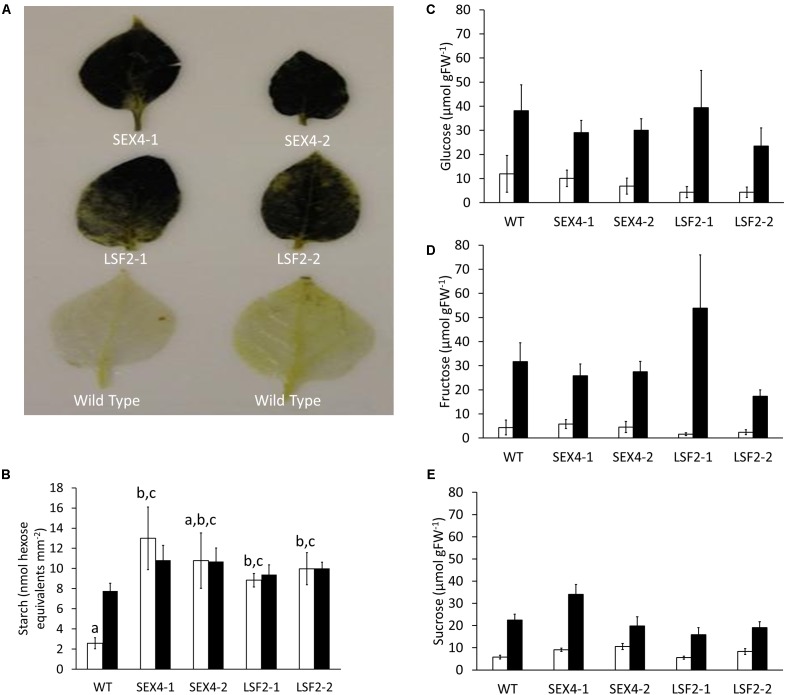
Starch degradation in leaves and tubers of transgenic lines. **(A)** Leaves of potato plants kept in the dark for 3 days before being bleached and stained for the presence of starch using iodine solution. **(B)** Starch present at the beginning (empty bars) and end (filled bars) of the day in leaves of the transgenic plants. **(C–E)** Glucose, fructose, and sucrose concentrations in detached tubers stored at room temperature (empty bars) or 4°C (filled bars) for 8 weeks. Data represents mean ± SEM of measurements from at least five individual tubers of each line. Letters represent groups with similar means at the 5% significance level as determined using the Bonferroni–Holm *post hoc* test following a one-way analysis of variance. Where no letters are present, no significant differences were found.

Staining of starch using Lugol’s solution in leaves that had been darkened for 72 h showed that the polymer was still present in all the transgenic lines, but not the control (**Figure 3A**). Quantitative analysis over a diel cycle demonstrated that leaves of three of the four transgenic lines contained significantly increased starch at the end of the dark period compared with the controls (**Figure 3B**). Tubers harvested from the plants were kept at 4°C for 8 weeks before glucose, fructose, and sucrose concentrations were determined. Although soluble sugars increased in tubers stored at 4°C compared with those stored at room temperature, no significant differences were found between the amounts of these sugars present in any of the lines (**Figures 3C–E**).

### Starch Granule Morphology

**FIGURE 4:**
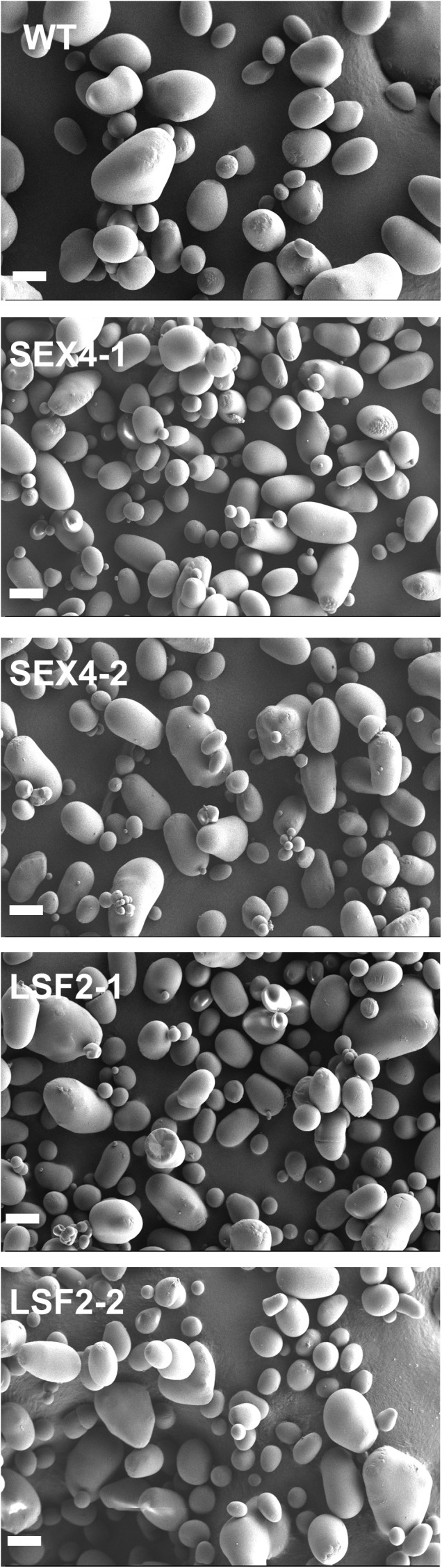
Purified tuber starch granules were imaged by scanning electron microscopy. Scale bar represents 20 μm.

Starch granules isolated from all the lines were visualized by scanning electron microscopy (**Figure 4**). This analysis demonstrated that the majority of granules from all lines were roughly ovoid, without any obvious morphological change between samples.

### Starch Chain Length Distribution

**FIGURE 5:**
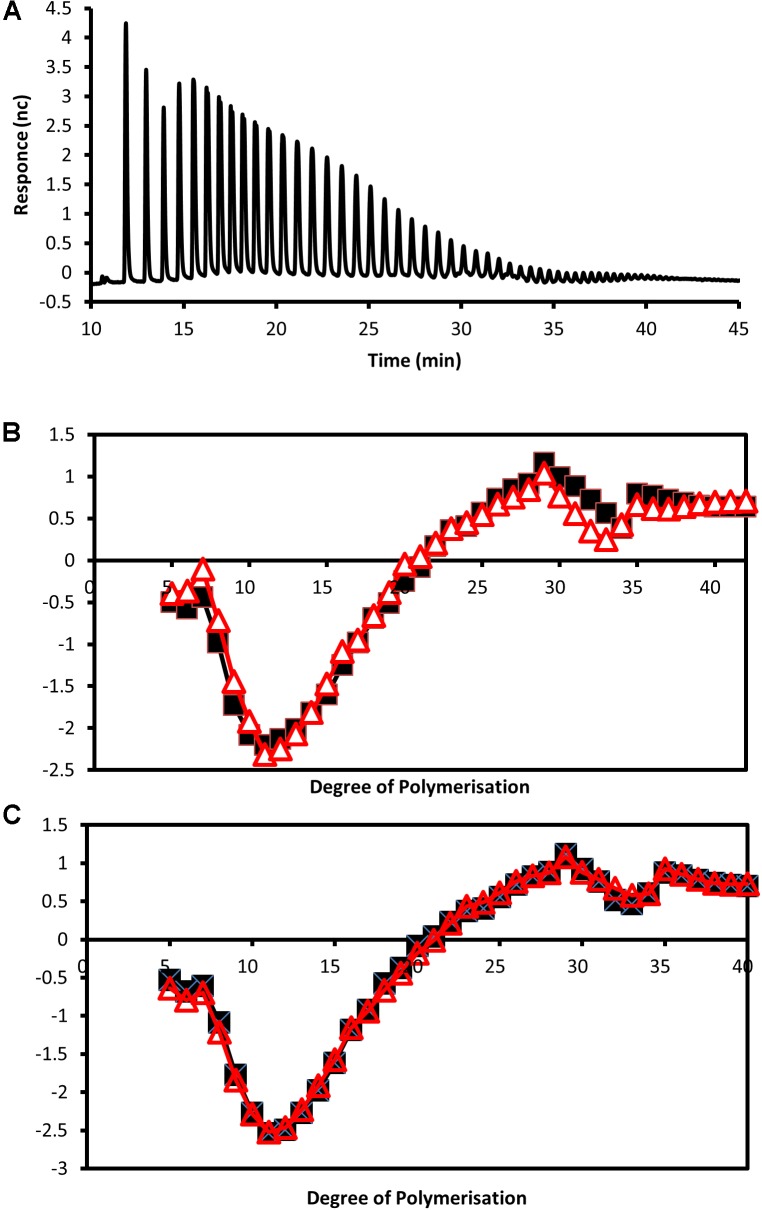
Analysis of debranched tuber starch by HPAEC-PAD. **(A)** Example of separation of isoamylase digested starch from a wild-type tuber by high-performance anion exchange chromatography. **(B,C)** Plots showing differences in detector response for chain lengths between approximately DP5 and DP40 of debranched starches from the wild type compared with starches from the transgenic lines with reduced expression of **(B)** *StSEX4* (black line with squares represent line SEX4-1 and red line with triangles SEX4-2) and **(C)** *StLSF2* (black line with squares represent line LSF2-1 and red line with triangles LSF2-2).

To examine if the genetic alteration also affected the branching distribution within starch, isoamylase digested samples were separated by HPAEC-PAD. It was found that starch from all the transgenic lines had significantly altered chain length distribution. Difference plots (**Figure 5**) demonstrated that the transgenic lines had fewer chains with degrees of polymerization (DP) between 5 and 20 and more of between DP20 and 40.

### Analysis of Tuber Starch

Some genetic manipulations affecting the structure of potato starch have been demonstrated to reduce starch accumulation, so we examined if starch content was altered in the transgenic tubers (**Table 1**). None of the transgenic lines contained significantly different amounts compared with the controls and the ratio of amylose to amylopectin was unaltered. However, ^31^P-NMR showed that the ratio of C6- to C3-bound phosphate was affected in the transgenic lines, with the amount of C6-bound phosphate being increased relative to C3-bound phosphate in the *StSEX4* repressed lines, while C3-bound phosphate increased in *StLSF2* repressed lines (**Table 1**). Measured amounts of C6-bound phosphate were also consistently and significantly increased in the *StSEX4* lines (**Table 1**). The ratios of phosphate bound at the C6 and C3 positions, determined by NMR, were used to calculate the amount of C3 bound phosphate in the different lines based on the measured amounts at the C6 position. In this case, a significant increase in C3-bound phosphate was found in both *LSF2* lines (**Table 1**). This led to an increase in total phosphate (combined C3 and C6) in all lines except *LSF2*-2 (**Table 1**). All the transgenic lines contained starch granules with a significantly reduced average diameter (**Table 1**).

**Table 1:** Characteristics of tuber starch from the potato lines.

Line	Tuber starch concentration (μmol hexose equivalents gFW^-1^)	Apparent amylose content (%)	Ratio of glucose 6-phosphate to glucose 3-phosphate	Glucose 6-phosphate content of starch (nmol G6P μmol hexose equivalents^-1^)	Glucose 3-phosphate content of starch (nmol G3P μmol hexose equivalents^-1^)	Glucose 3- and glucose 6-phosphate (nmol μmol hexose equivalents^-1^)	Average starch granule diameter (μm)	Swelling power (g g^-1^)
Wild type	609 ± 97	22.5 ± 2.0	2.03:1	14.9 ± 0.4^a^	7.3 ± 0.2^a^	22.2 ± 0.6^a^	39.42 ± 0.15^a^	19.4 ± 0.6^a^
SEX4-1	613 ± 26	23.1 ± 0.6	2.45:1	22.0 ± 0.5^b^	9.0 ± 0.2^b^	30.9 ± 0.8^b^	29.86 ± 0.08^b^	24.9 ± 0.9^b^
SEX4-2	797 ± 70	22.7 ± 1.7	2.71:1	18.5 ± 0.4^c^	6.8 ± 0.1^a^	25.3 ± 0.5^c^	29.68 ± 0.11^b^	21.8 ± 0.4^a,b^
LSF2-1	741 ± 87	21.5 ± 1.9	1.38:1	14.0 ± 0.2^a^	10.2 ± 0.2^c^	24.2 ± 0.4^a,c^	28.42 ± 0.06^c^	24.2 ± 0.6^b^
LSF2-2	656 ± 74	22.1 ± 0.6	1.55:1	16.4 ± 0.5^d^	11.4 ± 0.3^d^	27.9 ± 0.8^b^	26.11 ± 0.24^d^	25.8 ± 1.4^b^

*The data represent the mean ± SE of at least five measurements for the amount of starch, amylose and glucose phosphate determinations, and four measurements for the starch granule size which were performed on individual pooled samples of starch isolated from tubers of at least five plants. Letters represent groups with similar means at the 5% significance level as determined using the Bonferroni–Holm post hoc test following a one-way analysis of variance. Where no letters are present, no significant differences were found.*

### Starch Physical Properties

Because of these alterations in starch bound phosphate, chain length and granule size, we examined the swelling power of the starches. Three out of the four starches (isolated from lines *SEX4-1, LSF2-1, LSF2-2*) demonstrated significantly increased swelling power compared with the control (**Table 1**). To examine gelling in more detail we subjected the starches to analysis in a rapid viscoanalyzer (RVA). Peak viscosity was decreased in the LSF2 lines, while trough viscosity and breakdown were both decreased in starches from all the transgenic lines. On the other hand, final viscosity was significantly increased in all lines (**Table 2**).

**Table 2:** Viscosity of a starch/water slurry during heating and cooling in a rapid viscoanalyzer.

Line	Peak viscosity (cP)	Trough viscosity (cP)	Breakdown (cP)	Final viscosity (cP)	Setback (cP)
Wild type	21758 ± 608^a^	3392 ± 235^a^	18366 ± 844^a^	6374 ± 351^a^	2982 ± 586^a^
SEX4-1	21244 ± 161^a,b^	10084 ± 84^b^	11417 ± 245^b^	11417 ± 214^b^	771 ± 298^b^
SEX4-2	21832 ± 249^a^	7994 ± 251^c^	13838 ± 3^c^	8314 ± 276^c^	321 ± 25^b^
LSF2-1	17558 ± 103^c^	9986 ± 127^b^	7571 ± 231^d^	11918 ± 170^d^	1931 ± 42^a,b^
LSF2-2	19946 ± 131^b^	7399 ± 144^c^	12546 ± 13^b,c^	8327 ± 39^c^	928 ± 105^b^

*The data represent the mean ± SEM of two measurements. Letters represent groups with similar means at the 5% significance level as determined using the Bonferroni–Holm post hoc test following a one-way analysis of variance.*

## Discussion

This study was designed to assess the roles of SEX4 and LSF2 proteins in both starch degradation and starch phosphate metabolism in a plant storage organ, the potato tuber. Previous work has shown that mutations in the genes encoding these proteins affects leaf starch metabolism, and can increase amounts of starch bound phosphate (Kötting et al., 2009; Santelia et al., 2011). It is unclear, however, whether repression of orthologous genes would also lead to higher levels of starch phosphorylation in storage organs. Several studies have identified biotechnological methods that can increase starch bound phosphate in cereal endosperm and potato tuber (Abel et al., 1996; Safford et al., 1998; Jobling et al., 1999; Schwall et al., 2000; Carciofi et al., 2011; Du et al., 2012; Chen et al., 2017; Xu et al., 2017a,b), although none did so through repression of either *SEX4* or *LSF2*. Therefore, manipulation of these genes has the potential to expand the known methodologies that can be used to improve starches for industrial use.

To examine this we manufactured transgenic plants repressed in transcription of potato orthologs of either *SEX4* or *LSF2* (**Figure 1A**). There were no obvious differences in plant morphology (**Figure 2A**) and total tuber production was not consistently altered in the transgenic lines (**Figure 2B**). However, there was a significant decrease in average tuber weight in both SEX-4 lines and one of the LSF2 lines (**Figure 2C**) indicating that repression of *StSEX4* or *StLSF2* affect tuber initiation. Leaf starch degradation was affected in both *StSEX4* and *StLSF2* repressed lines. Qualitative iodine-staining showed that leaves kept in the dark for 3 days still contained significant amounts of starch (**Figure 3A**). Quantitative analysis demonstrated that leaves from both *StSEX4* and *StLSF2* repressed lines contained significantly more starch at the end of the night than wild type plants (**Figure 3B**). Thus, both genes are involved in leaf starch degradation in potato. Furthermore, *StLSF2* seems to play a bigger role in leaf starch degradation in potatoes than the orthologous gene does in Arabidopsis, since *Atlsf2* knockout mutations have nearly normal rates of starch degradation (Santelia et al., 2011). Despite the clear effect of both genes on starch degradation in potato leaves, repression of neither gene affected the amount of reducing sugars that accumulated in tubers stored at 4°C (**Figures 3C–E**). One potential explanation for this would be that starch degradation occurs continuously at a lower rate in cold-stored tubers than in leaves, where almost all starch present needs to be degraded during the night period prior to the commencement of starch synthesis the next morning. It is possible that removing SEX4 or LSF2 may, therefore, be less effective at repressing this process in tubers simply due to the rate of degradation within these different tissues. Alternatively it may indicate that functional redundancy is more important in this step of the starch degradation pathway in potato tubers than in leaves. A final possibility for the lack of effect on cold-induced sweetening is that the pathway of starch degradation established in leaves does not represent the one present in tubers. This process needs to be investigated in more detail.

Many studies have focused on the influence of starch phosphate on alterations in physical properties through analysis of starches from different botanical origins, different plant varieties where phosphate contents have been altered (Blennow et al., 2000; Noda et al., 2004, 2007; Zaidul et al., 2007b,d), or genetically modified plants with altered starch phosphate (Lorberth et al., 1998; Chen et al., 2017; Xu et al., 2017a,b). Covalently bound phosphate has been identified as an important component affecting some physical properties of starch, such as solubility and swelling power (Sitohy et al., 2000; Zaidul et al., 2007a). In addition, it is thought to make starch less enzymatically degradable in the gut (Wickramasinghe et al., 2009; Lu et al., 2012), possibly leading to improved effects on human health and metabolism when ingested in the diet (Kanazawa et al., 2008).

In this study, we successfully increased total starch phosphate (combined C6- and C3-bound) by 9% in line LSF2-1 and up to 39% in line SEX4-1 (**Table 1**). Within the *StSEX4* repressed lines, the main phosphate increase was at the C6 position, while in the *StLSF2* repressed lines it was at the C3 position (**Table 1**). This corresponds to the known roles of the two enzymes encoded by the orthologous Arabidopsis genes where AtSEX4 and AtLSF2 are thought to have greater specificity for removing phosphate from the C6 and C3 positions, respectively (Kötting et al., 2009; Santelia et al., 2011). Mutations in *AtLSF2*, but not *AtSEX4*, lead to increases in starch bound phosphate, due to alterations in the amounts bound at the C3 position (Santelia et al., 2011). Although mutation of *AtSEX4* does not increase starch bound phosphate, it does lead to increased accumulation of phosphorylated malto-oligosaccharides in the stroma (Kötting et al., 2009). There are several potential reasons why starch bound phosphate was increased in the *StSEX4* RNAi repressed lines, while no such increase was observed in leaf starch of an *Atsex4* mutant. Firstly, it may be due to differences in starch turnover between leaves and tubers. Starch is synthesized and broken down every day in leaves, while in tubers it accumulates over a period of several months. If SEX4 removes a small amount of phosphate from the surface of the granule on a daily basis, absence of the protein would lead to a much larger increase in starch phosphate in tubers than leaves simply due to the greater amount of starch that accumulates. Secondly, StSEX4 may be more effective at removing starch bound phosphate than AtSEX4, or tuber starch could be a better substrate than leaf starch for SEX4 proteins, so that the absence of StSEX4 has a greater effect. Finally, the environment within a potato tuber plastid is different from a chloroplast. AtSEX4 has been shown to associate with the starch granule in the light and dissociate in the dark. This dissociation is thought to be triggered by increases in pH and by oxidizing conditions (Sokolov et al., 2006). The stroma in potato tuber amyloplasts would be unlikely to undergo such dramatic changes in redox and pH as occurs during a day/night cycle in a chloroplast, so it may be that a large proportion of StSEX4 is constantly associated with the surface of amyloplast starch granules and affects the amount of granule-bound phosphate.

Differences in starch phosphate concentrations have been reported to be accompanied by alterations in the branching structure of the starch polymer as a strong correlation between the chain length distribution and phosphate content of starches from different botanical origins has been identified (Blennow et al., 2000). Elevated starch bound phosphate in potato and rice caused by increased GWD is accompanied by altered chain lengths (Chen et al., 2017; Xu et al., 2017a). In the potato study, the starch contained an increase in chains of DP6 and decreases of chains between DP8–12 and DP16–35, but only when compared to lines transformed with the same construct that demonstrated decreased starch phosphate (Xu et al., 2017a). High phosphate rice starch showed decreases in chains of DP14 and DP15, with increases in chains greater than DP37 (Chen et al., 2017). In the current study, the enzymes that were repressed are involved in dephosphorylating starch, and so cannot alter the branching of the starch polymer directly. Nevertheless, we found that transcriptional repression of either gene resulted in a decrease in the degree of branching with fewer chains between DP5–20 and more long chains between DP20–40 (**Figure 5**). The structural alterations caused by changes in bound starch phosphate may affect the starches suitability as a substrate for enzymes that determine the chain length of the polymer. Although starch branching enzymes are known to be able to use phosphorylated glucans as substrates (Viksø-Nielsen et al., 1998), it isn’t clear if the amount of phosphate present – and its position on the glucan chain – affects their activity. Intriguingly, repression of potato starch branching enzymes and one starch synthase isoform is known to increase the phosphate content of starch (Abel et al., 1996; Safford et al., 1998; Jobling et al., 1999; Schwall et al., 2000; Hofvander et al., 2004; Wischmann et al., 2005; Du et al., 2012) indicating an interplay between these enzymes and starch bound phosphate. Although neither GWD nor PWD were increased in the transgenic lines (**Figure 1B**), it is still possible that the alteration in chain length may also have led to increased phosphate incorporation by GWD as this enzyme has been shown to have a higher preference for chains between DP 28–30 (Mikkelsen et al., 2004), which are present in increased numbers in starches from the transgenic lines (**Figure 5**). This would, however, only help to explain the increased phosphate in the SEX4 lines as GWD incorporates phosphate specifically at the C6 position (Ritte et al., 2006).

The increase in starch phosphate affected starch granule size, with all the transgenic lines producing granules that were reduced in diameter (**Table 1**). This was not caused by a reduction in starch accumulation within the tuber (**Table 1**) and so must be due to an effect either on granule initiation or size determination, processes which are poorly understood (Pfister and Zeeman, 2016). It appears unlikely that the change in starch phosphate alone leads to this alteration as increases in starch phosphate, through expression of a mammalian *Laforin* gene, led to starches with an increased average granule diameter in potato tubers (Xu et al., 2017b). A number of polypeptides have been demonstrated to impact starch granule initiation (Bustos et al., 2004; Roldan et al., 2007; Szydlowski et al., 2009; Crumpton-Taylor et al., 2013; Matsushima et al., 2014, 2016; Peng et al., 2014; Seung et al., 2017; Lu et al., 2018) and, in this case, we hypothesize that the increase in starch phosphate and/or chain lengths alters the substrate that these enzymes use in such a way as to lead to the reduced granule size.

The alteration in size was not accompanied by an obvious change in granule morphology (**Figure 4**). Two recent studies using potato have led to plants synthesizing tuber starch with altered starch phosphate. Xu et al. (2017a) reported that plants over-expressing a *GWD* gene, showed altered starch granule morphology only in lines where starch phosphate was reduced, not where it was increased. In the second study (Xu et al., 2017b), starch phosphate was increased through expression of a mammalian *Laforin* gene, and granules from these plants exhibited both irregular shapes and surface. Our data indicate that the alteration in granule morphology identified by Xu et al. (2017b) is unlikely to be caused by the increase in starch bound phosphate that they engineered since we would then have expected to observe similarly shaped granules in our lines.

The alterations that we identified in starch isolated from the transgenic plants led us to examine its physical properties. As the presence of covalently bond phosphate is known to improve starches hydration capacity (Jobling, 2004) we examined its swelling power and found a significant increase for this parameter in the starches from three out of four of the transgenic lines (**Table 1**), demonstrating increased water binding. Starch phosphate has also been positively correlated with peak viscosity and breakdown, and negatively correlated with setback (Noda et al., 2004; Karim et al., 2007; Zaidul et al., 2007b,c). As would be predicted from those studies, our rapid viscoanalyzer data show that starches from three of the high phosphate lines have lower setback. However, we did not observe the expected increases in either peak viscosity or breakdown in the high phosphate starches (**Table 2**). The reasons for these differences are unclear. Although starch phosphate is increased in the transgenic lines, we also observed changes in chain lengths within the starch molecules and granule size. As RVA examines the sum of the effects that all these structural changes have on the starches physical properties, it is possible that these other alterations have the opposite effect on peak viscosity and breakdown to that caused by increased starch phosphate.

The alterations that we have identified in starches from the transgenic lines indicate that they may have improved properties for use in specific industries. The observed increased final gel viscosity would be advantageous in the food industry as less starch would be required to achieve the same thickening effect. Alterations in starch granule size has also been shown to help in the manufacture of noodles, with smaller granules – as found in the transgenic line – being preferred (Chen et al., 2003). Finally, increased starch bound phosphate is thought to contribute to the stickiness of starch gels, a property which is advantageous to the paper industry (Blennow et al., 2003). This study has, therefore, identified a new method that can be used to biotechnologically improve starches for industrial use. High phosphate starches have been associated with decreased digestibility by amylolytic enzymes (Kanazawa et al., 2008), which is thought to be advantageous in mitigating type II diabetes as glucose release into the bloodstream occurs over a longer period after ingestion (Miao et al., 2015). We wish, therefore, to examine these starches in future animal-feeding studies to examine potential health benefits.

## Author Contributions

ES transformed the plants and performed analysis on immunoblots, RVA and cold-sweetening. JJ and ES analyzed starch phosphate, starch granule size, and cold-sweetening. BL performed the semi-quantitative RT-PCR. KO generated the phylogenetic tree. GG and SZ produced the chain length distributions of debranched starch. FD was responsible for the ^31^P NMR. CvdV supervised the plant transformation. JK and JL designed the project while JL and ES wrote the paper together. All authors proofread the paper and provided feeback.

## Conflict of Interest Statement

The authors declare that the research was conducted in the absence of any commercial or financial relationships that could be construed as a potential conflict of interest.

## Footnotes

1 www.aaf-eu.org/european-starch-industry

2 https://blast.ncbi.nlm.nih.gov/Blast.cgi

3 http://cnrgv.toulouse.inra.fr/en

4 http://solanaceae.plantbiology.msu.edu

## Supplementary Material

FIGURE S1 Phylogenetic analysis of SEX4, LSF1, and LSF2 DSP domains. Parsimony bootstrap tree using 100 replicates. *Arabidopsis, Nicotiana benthamiana*, and *Solanum tuberosum* sequences for SEX4, LSF1, and LSF2 are indicated with a star (⋆) within the respective groups (shaded). All other species are either putative orthologs of SEX4, LSF1, and LSF2 in higher plants, or outliers containing DSP domains as described in Santelia et al. (2011).
