# Supplementary figures and images for: Repression of Sex4 and Like Sex Four2 Orthologs in Potato Increases Tuber Starch Bound Phosphate With Concomitant Alterations in Starch Physical Properties
Source: Front Plant Sci. 2018 Jul 23;9:1044. doi: 10.3389/fpls.2018.01044 (PMC6064929; doi:10.3389/fpls.2018.01044)

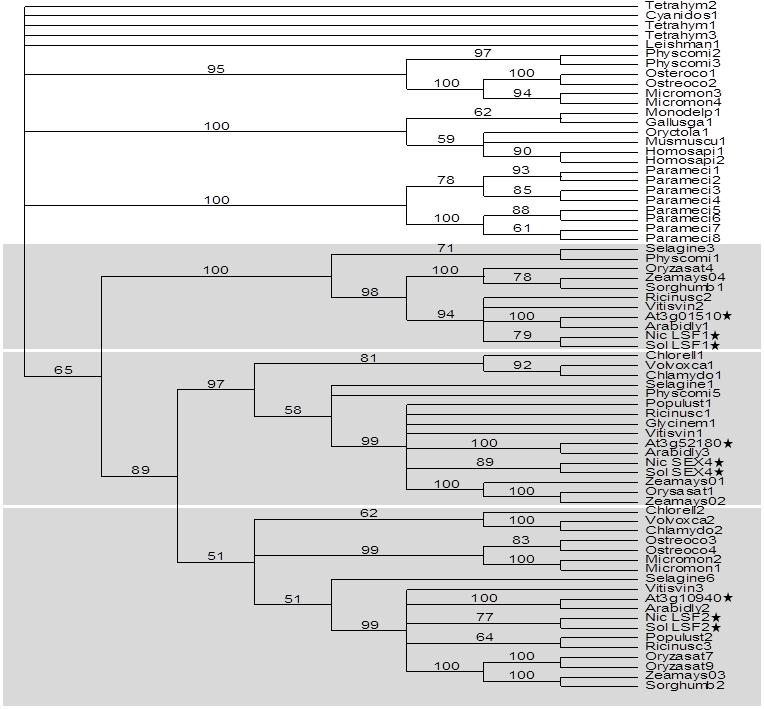

Supplement: FIGURE S1 — Phylogenetic analysis of SEX4, LSF1, and LSF2 DSP domains. Parsimony bootstrap tree using 100 replicates. Arabidopsis, Nicotiana benthamiana, and Solanum tuberosum sequences for SEX4, LSF1, and LSF2 are indicated with a star (⋆) within the respective groups (shaded). All other species are either putative orthologs of SEX4, LSF1, and LSF2 in higher plants, or outliers containing DSP domains as described in Santelia et al. (2011). [file Image_1.TIF]
